# Stress and Obesity: Are There More Susceptible Individuals?

**DOI:** 10.1007/s13679-018-0306-y

**Published:** 2018-04-16

**Authors:** Eline S. van der Valk, Mesut Savas, Elisabeth F. C. van Rossum

**Affiliations:** 1 Obesity Center CGG, Erasmus MC, University Medical Center Rotterdam, Room D-428, P.O. Box 2040, 3000 CA Rotterdam, The Netherlands; 2 Department of Internal Medicine, division of Endocrinology, Erasmus MC, University Medical Center Rotterdam, Rotterdam, The Netherlands

**Keywords:** Obesity, Stress, Cortisol, Hair cortisol, Glucocorticoid receptor, Glucocorticoid receptor polymorphisms, Corticosteroids

## Abstract

**Purpose of Review:**

Stress has long been suspected to be interrelated to (abdominal) obesity. However, interindividual differences in this complex relationship exist. We suggest that the extent of glucocorticoid action partly explains these interindividual differences. We provide latest insights with respect to multiple types of stressors.

**Recent Findings:**

Increased long-term cortisol levels, as measured in scalp hair, are strongly related to abdominal obesity and to specific mental disorders. However, not all obese patients have elevated cortisol levels. Possibly, the interindividual variation in glucocorticoid sensitivity, which is partly genetically determined, may lead to higher vulnerability to mental or physical stressors. Other evidence for the important role for increased glucocorticoid action is provided by recent studies investigating associations between body composition and local and systemic corticosteroids.

**Summary:**

Stress may play a major role in the development and maintenance of obesity in individuals who have an increased glucocorticoid exposure or sensitivity. These insights may lead to more effective and individualized obesity treatment strategies.

## Introduction

Obesity is a rapidly increasing pandemic with major consequences for public health. In the past years, evidence is mounting that stress, and particularly, an increase of the glucocorticoid stress hormone cortisol plays a role in the development of obesity. However, it seems that not all individuals respond to stress in the same way. This poses the question whether there is interindividual variation in the biological response to stress. In this review, we will explain how individual susceptibility to stress and obesity is determined, with a focus on cortisol, one of the main hormones involved in the biological stress response. Cortisol, a glucocorticoid (GC) hormone, is known to cause a redistribution of white adipose tissue to the abdominal region and in addition increases appetite with a preference for energy-dense food (“comfort food”) [1•]. Patients who are chronically exposed to high levels of glucocorticoids, like in Cushing’s syndrome or when using high doses of exogenous GC, develop abdominal obesity, metabolic syndrome (MetS), and eventually cardiovascular diseases (CVD).

Interestingly, in our modern society, the obesity pandemic coincides with an increase in factors that enhance cortisol production, such as chronic stress, consumption of food with a high glycemic index, and a reduced amount of sleep [2, 3]. This suggests a vicious circle, where increased glucocorticoid action, obesity, and stress interact and amplify each other [4]. This hypothesis is supported by recent studies demonstrating significant correlations between obesity and long-term cortisol levels, as measured in scalp hair, in both adults [5, 6••, 7] and children [8]. This article will briefly discuss this novel technique of cortisol measurement and its implications in stress-related research in the past decade and in the future [9]. Additionally, it will focus on the mechanisms and conditions that influence the sensitivity to GC at a molecular level.

This review will also provide a short overview of recent developments regarding several types of stress (e.g., acute/chronic stress, mental/physical stress but also exposure to exogenous stress hormones) and their relationships with obesity. In addition, existing literature explaining interindividual differences in these complex relationships will be discussed. All these concepts are then integrated in a conceptual model with obesity, chronic stress, and increased glucocorticoid action as its key components.

## Stress

Stress, both physical and psychological, can be seen as a part of life that every individual will experience to some extent. Goldstein described stress as a condition in which expectations, whether genetically programmed, established by prior learning, or deduced from circumstances, do not match current or anticipated perceptions of the internal or external environment [10]. This mismatch between what is observed or sensed and what is expected or programmed evokes patterned, compensatory responses. This assembly of responses is generally called the “stress response” and applies to mental stress. From a biological perspective, there are also other types of stressors, such as sleep deprivation, pain, inflammation, or use of exogenous glucocorticoids (GCs), which can all elicit a stress response at the cellular level.

### The Acute Stress Response

Within seconds after a (perceived) stressor, catecholamines such as epinephrine and norepinephrine (associated with the fight/flight response) are produced in the sympathetic nervous system and in the adrenal medulla. They increase heart rate and stroke volume and cause vasoconstriction of blood vessels in the skin and the gut. Furthermore, epinephrine stimulates glycogenolysis in the liver, leading to higher serum glucose levels providing energy for a defensive reaction [11]. Peripherally produced epinephrine and norepinephrine do not cross the blood-brain barrier. Within the brain, the locus coeruleus produces norepinephrine and supports arousal, vigilance, and narrowing of attention [12••].

Catecholamines are also linked to the hypothalamic-pituitary-adrenal (HPA) axis with its end-product cortisol, which responds within minutes to hours and supports the action of catecholamines. Following an *acute stressor*, the release of corticotropin-releasing-hormone (CRH) is initiated from neurons in the parvocellular division of the paraventricular nucleus (PVN) [12••, 13]. This leads to a rise in adrenocorticotropic hormone (ACTH), which is secreted in a pulsatile way after initiation by CRH [12••]. The CRH release depends on the stressor’s duration, intensity, and feedback. ACTH subsequently stimulates the production of GCs from cholesterol and other steroid precursor hormones in the adrenal glands.

CRH suppresses appetite acutely after stress [14], probably by inhibiting food intake that is stimulated by neuropeptide Y (NPY) [15]. However, once extra GCs are produced, the intake of food is stimulated [15, 16], which occurs already as soon as 1 h after injection of CRH [17]. GCs stimulate the actions of NPY and reduce the sensitivity of the brain to leptin and to insulin by enhancing insulin resistance. The latter also inhibits feeding-stimulatory pathways in the brain [15]. Moreover, GC specifically increase a preference for food that is rich in fat and sucrose [1•, 15]. An acute stress response ends when the stressor disappears. At this point, CRH release from the PVN is inhibited by several mechanisms that are mainly driven by cortisol, thereby reducing its own production [12••]. The glucocorticoid receptor (GR) is crucial for this negative feedback loop [12••]. The rapid termination of CRH secretion and subsequent decrease in ACTH release causes GC secretory activity to gradually return to baseline [12••], thereby ending the stress response.

The increased secretion and effects of these major stress mediators that are induced by acute stress, though essential for the survival of an organism, can also have negative effects. Excessive sympathetic activity may lead to hypertensive situations whereas increased parasympathetic action may lead to hypotensive situations. In this way, angiokinetic phenomena such as migraine may develop [18]. Furthermore, the CRH that is involved in acute stress can induce degranulation of mast cells in specific organs, thereby eliciting asthma or eczema. Also, acute stress can lead to different types of pain, gastrointestinal symptoms, and mental disturbances such as panic attacks and psychoses [18].

### Chronic Stress

The main effectors in *chronic stress* are GC, such as cortisol. The effects of GC are, at a cellular level, exerted through two intracellular receptors, the mineralocorticoid receptor (MR) and the glucocorticoid receptor (GR). Although the MR has a stronger affinity for GCs, the presence of 11-beta-hydroxysteroid dehydrogenase type II (11βHSD2), which locally converts cortisol to inactive metabolites, prevents a glucocorticoid action in various tissues, such as the kidneys and certain brain areas [19].

When GC bind to the GR, this receptor will translocate into the nucleus, where it influences gene expression [20]. GRs are widely distributed throughout the brain and peripheral tissues; thus, glucocorticoids are involved in a variety of processes. They regulate cardiovascular tone; serve as an intermediary in metabolism through catabolic actions that take place in the liver, muscle, and adipose tissue; and they impact the inflammatory and immune response [21]. In addition, GCs influence essential functions such as reproduction, growth, behavior, water and electrolyte balance, and cell proliferation and survival [13].

The biological effects of increased levels of endogenous cortisol have a substantial overlap with the effects of chronic exposure to exogenous synthetic glucocorticoids, as they exert their biological actions through the same receptor [22••]. However, synthetic glucocorticoids are not bound to cortisol binding globulin in the plasma and are not metabolized by 11βHSD2 [22••].

## Consequences of Chronic Stress

### Mental Factors Related to Chronic Stress and HPA Hyperactivity

It is known that chronic stress is linked to several behavioral and neuropsychiatric conditions. Regarding the exact pathophysiological mechanisms, not only speculations have been made about the effect of CRH [23], but also its end product cortisol has been hypothesized to have a pathophysiological role in stress-related mental disorders such as anxiety and depression [24, 25]. Currently, the relatively novel technique of hair cortisol concentration (HCC) measurement [26] has also been implemented in research of mental disorders and conditions that are related to stress [27]. This unique and recently developed and optimized technique measures long-term cortisol levels using scalp hair [9, 26] and thus overcomes the limitations of serum, saliva of urine cortisol analysis that represents “snapshots” of cortisol levels instead of a consistent measurement of chronic cortisol levels. Studying the correlation between mental states and traits and chronic cortisol levels is important, as this may be an important determinant in the vicious circle of stress and obesity [28•].

Recently, Stalder et al. reported in a meta-analysis that perceived chronic stress is associated with an estimated 22% elevated HCC. Particularly, groups who had stress ongoing at the time of study exhibited a 43% increase of HCC [6••]. Also, hypercortisolism was found in scalp hair in patients reporting severe symptoms of burnout [29]. Interestingly, Jackson et al. recently found that obese individuals who experience discrimination that is explicitly weight-related exhibit higher HCC levels than obese persons who did not feel discriminated because of their weight. This effect was not present for discrimination in general [30]. In this way, weight discrimination by itself may contribute to progression of the obese state by increasing cortisol levels.

Furthermore, Karlen et al. found that children with more psychosocial exposures during pregnancy and early life had higher HCC levels [31]. It was also found that a lower family income [32] and lifetime exposure to trauma [33] were associated with a higher HCC. In adults, however, a past trauma corresponded with lower HCC [6••, 34–36], whereas in the acute phase, cortisol levels were raised [37]. For example, a recent study shows that permanently staying asylum seekers have lower HCC than the reference group whereas recently fled asylum seekers have higher HCC, but no differences were found between the recently fled asylum seekers with post-traumatic stress disorder (PTSD) and those without PTSD [38]. Possibly, a traumatic experience temporarily raises systemic cortisol levels, which fade over the course of time leading to subsequent lower HCC.

Regarding other mental disorders, it has been challenging to prove a consistent relation between HCC and mood disorders such as depression and bipolar disorder [6••, 29]. For the diagnosis of depression, some studies reported higher HCC compared to controls [39, 40], whereas others found no association [41, 42] or lower HCC [43, 44]. Regarding depressive symptoms, strong correlations with cortisol have been described [45, 46], whereas others found no association [47, 48]. The use of diverse populations with relatively small sample sizes may be accountable for these differences, as well as the duration of symptoms. However, large study comprising 921 participants with a current or remitted depressive and/or anxiety disorder confirmed a significant association with the presence of a comorbid depressive and anxiety disorder. In this study, the severity of depressive symptoms and anxiety symptoms was also correlated to HCC (Sabine M. Staufenbiel, Brenda W.J.H Penninx, Lotte Gerritsen, Albert M. van Hemert, Gerard Noppe, Yolanda B. de Rijke, Elisabeth F.C. van Rossum, unpublished results 2018). The use of antidepressants may complicate this research, as they could influence the HPA axis, possibly by modulating the GR [49]. Specifically, selective serotonin reuptake inhibitors (SSRIs) are associated with higher HCC in most studies ([50–52]. As depression and obesity are strongly related in a bidirectional manner, unraveling the shared pathophysiological mechanisms is of profound interest for future combined treatment strategies [28•].

### Physical Consequences of Chronic Stress

Chronic stress causes an elevated baseline HPA-activity and increased HPA axis responsiveness [12••, 53]. In this case, the prolonged effects of the effector molecules of the stress system, including cortisol, norepinephrine, and CRH, can lead to disorders in the aforementioned target tissues, including a broad spectrum of inflammatory, metabolic and neuropsychiatric diseases [13]. As for the immune system, stress has complex actions. GCs alter the function of leukocytes and other immune cells, and decrease proinflammatory cytokines. Also, GCs as well as catecholamines induce a switch from Th1 to Th2 cells, which increases the risk for auto-immune disorders such as systemic lupus erythematosus, Graves’ disease, and allergic conditions [13]. On the contrary, pro-inflammatory cytokines have a stimulatory effect on the stress system, which leads to increased GC levels that again suppress the inflammation [18]. Endocrine consequences following the increased effect of GCs include inhibition of the growth hormone axis, thyroid axis, and gonadal axis, contributing to the loss of muscle mass and bone mass and visceral fat accumulation that is seen in increased GC exposure [13]. The subsequent visceral obesity and loss of muscle mass are associated with clinical parameters comprising the MetS: dyslipidemia and hypertension and type 2 diabetes mellitus, which can ultimately lead to cardiovascular diseases [1•].

As mentioned previously, exogenous synthetic GCs exert roughly the same biological effects as glucocorticoids from the endogenous pathway [22••]. It is therefore not surprising that in clinical practice, associations between the use of synthetic corticosteroids and obesity and the metabolic syndrome are found. Interestingly, obese patients visiting an outpatient clinic for obesity had used corticosteroids in the last 3 months nearly twice as often as non-obese controls [54]. We also observed in a large sample of more than 140,000 adult individuals from the general population that use of both systemic and the local types was associated with the presence of metabolic syndrome, as well as an higher BMI. However, causality still has to be proven. Suggestive of a causal relation is the meta-analysis of Broersen et al., showing that local corticosteroids have systemic effects such as adrenal suppression [55, 56]. The effects of chronically elevated corticosteroid levels have been shown to be modified by genetic variation in the GR gene [22••]. Individuals carrying GR gene polymorphisms which are associated with a relative hypersensitivity seem to be more vulnerable for the adverse effects such as higher fat percentages, higher serum leptin, and an adverse cardiometabolic risk profile [57]. This will be further discussed below.

If future research will show that the relation between obesity and corticosteroid use is causal, it may have large consequences for public health, as a substantial part of the general population uses these (local) corticosteroids (nearly 11% in the approximately 140,000 participants of the population-based Lifelines cohort study) [58]. Furthermore, if we consider the individual differences in the biological effects of these agents, it seems that specific persons can experience a considerable weight gain after initiation of these agents.

## Novel Insights in the Relation Glucocorticoid Exposure and Obesity

### Cushing’s Syndrome as a Model of a Pathological Status of Stress and Obesity

In the past years, an imbalance between energy intake and energy expenditure was regarded the main cause of the obesity pandemic. However, considering the abovementioned effects of chronic cortisol exposure, evidence is mounting that cortisol is also a kingpin in this pandemic. The effects of long-term exposure to extremely elevated levels of cortisol are demonstrated by Cushing’s syndrome (CS). This is a condition characterized by pathologically elevated cortisol levels, usually caused by a pituitary or adrenal adenoma. In CS, the excess of cortisol can lead to a variety of clinical manifestations, such as abdominal obesity, hypertension, abnormal glucose tolerance, and proximal muscle weakness [59]. The striking resemblance of CS patients and the more common MetS suggests a causal role for GCs. Importantly, treatment can to some extent reverse the adverse metabolic and anthropometric characteristics, although some may remain [60]. This, at least partial, reversibility of the CS indicates that this presumed hypercortisolism could provide promising future treatment opportunities for the metabolic syndrome.

However, when studying the relation between GC and human obesity, conflicting results were found with respect to cortisol levels in obesity [61–64]. The diagnostic tests that were previously used, for measuring cortisol in serum, saliva or urine, are difficult to interpret when applied to measure subtle hypercortisolism in “common” obesity. This is mainly due to the circadian rhythm of cortisol, its pulsatile secretion, and relatively short half-life in plasma (approximately 66 min) [65], as well as daily variations due to changing circumstances like acute stress. As previously mentioned, scalp hair analysis of cortisol overcomes these limitations.

### Correlations Between Long-Term Cortisol and Obesity

It has been demonstrated that, on average, obese individuals have higher hair cortisol levels [7] The meta-analysis of Stalder et al. also found consistent evidence for a relationship between HCC and BMI (9.8% increase of hair cortisol for each 2.5-point increase in BMI) [6••]. Afterwards, several cohort studies have confirmed the relationship between adiposity measures and HCC in adults [5, 66]. Interestingly, also in a large cohort study of more than 3000 6-year-old children, those with the highest hair cortisol concentrations had an almost 10-fold increased risk of obesity [8]. In the latter study, cortisol was specifically correlated to an increase of abdominal fat mass, which is similar to the effect of cortisol in CS. In addition to these cross-sectional findings, HCC also correlated to a more persistent obesity over time [5]. So, on average, long-term cortisol levels are elevated in obese individuals, and seem in particular related to increased abdominal fat mass. This specific distribution may be explained by the greater density of GRs in visceral adipose tissue than in other adipose tissues, causing fat redistribution to the abdominal region [1•, 67]. This could be further enhanced by an increased expression of 11βHSD1 leading to more active cortisol [68].

### Potential Mechanisms and Treatment Targets of Elevated Cortisol in Obesity

This brings up the question what causes the increased chronic cortisol levels in the majority of patients with obesity and whether there are more susceptible individuals. Despite the elevated average chronic cortisol levels in obesity, not all individuals with obesity have higher HCC. When taking a closer look at the sample of obese individuals, it can be observed that, within the obese group, roughly half of the obese patients appear to be *normocortisolistic*, whereas the others can be categorized as *hypercortisolistic* [7]. One can be speculate that hypercortisolistic obesity may represent more abdominal obesity and is more tightly linked to the metabolic syndrome and cardiovascular diseases. This concept is supported by studies showing that persons with the highest hair cortisol had the highest risk of metabolic syndrome [69] and cardiovascular diseases [70]. It is conceivable that these “hypercortisolistic” obese persons could benefit more from cortisol reducing treatment strategies than “normocortisolistic” obese individuals. Studies to elucidate this issue are ongoing.

There are several mechanisms that can potentially contribute to higher cortisol levels in individuals with obesity. First of all, there could be an overactivity of the HPA axis, including increased CRH and ACTH. This could be due to the intake of food with a high glycemic index [71], chronic stress [72], chronic pain [73], alcohol [74], and chronic sleep deprivation [75]. HPA activity also seems higher in patients with the night eating syndrome [76], although it is not known whether the actual timing of meals may influence the HPA axis. Secondly, inflammatory markers such as interleukin-6, TNF-alpha, and IL-1β can influence the HPA axis [77]. Thirdly, it is conceivable that the different cortisol levels may be due to individual variation in enzymes that are involved in cortisol metabolism, such as 11-β-hydroxysteroid dehydrogenases enzymes type 1 and 2 (11βHSD1 and 11βHSD2). The enzyme 11βHSD1 catalyzes the regeneration of the inactive cortisone to its active form cortisol. It was found to have a significantly higher expression in adipose tissue of obese individuals versus fat tissue of non-obese individuals, both in men and in women in most studies [64]. In the liver of obese patients, however, the activity of 11βHSD1 seems to be decreased [64, 78]. Moreover, in obesity, there may also be other enzymes responsible for altered cortisol levels or altered effects of cortisol, that include 5α and 5β reductases [78] and bile acids [79]. Also, severe liver steatosis could play a role, as the activity of hepatic enzymes responsible for cortisol clearance and regeneration is altered in patients with hepatic steatosis [80].

In the past years, several studies have investigated the potential of 11βHSD1 inhibitors and found modest effects on glycemic control in patients with type 2 diabetes mellitus, however not enough to compete with existing medication [81, 82]. The effect on other features of the metabolic syndrome, including hypertension, dyslipidemia, and obesity, was thus far also modest [82]. It is conceivable that these agents could be more potent if they are used selectively in *hypercortisolistic* patients with obesity. Future studies need to confirm this hypothesis.

Also, GR antagonists, such as mifepristone, lowered fasting glucose and insulin levels when given simultaneously with metyrapone in men with type 2 diabetes [83]. Other potential strategies to target hypercortisolism include non-pharmacological options such as mindfulness, which has been studied in patients with structural heart disease [84].

## Glucocorticoid Sensitivity at a Molecular Level and the Relation with Obesity and Stress-Related Disorders

### The Glucocorticoid Receptor

The clinical effects of glucocorticoids are determined not only by their quantity, but also by the individual sensitivity to glucocorticoids at a tissue level [85]. Cases of extreme glucocorticoid sensitivity, such as primary generalized glucocorticoid hypersensitivity, and of its opposite, extreme glucocorticoid resistance (Chrousos syndrome) have been described [86]; however, there are also variations within the normal range. A key player in glucocorticoid action is the GR, also known as nuclear receptor subfamily 3, group C, member 1, or NR3C1. When cortisol binds to the GR, it becomes activated and moves into the nucleus. In the cytoplasm, a multiprotein complex, which is formed by several folding chaperone proteins (heat shock proteins and FK506 binding proteins, FKBPs), regulates the activity of the GR [87]. The GR can activate or repress transcription of target genes through several mechanisms, which include direct interaction with the glucocorticoid-responsive elements in the promoter regions of target genes, or interaction with other transcription factors thereby influencing gene expression indirectly [88]. Thus, the individual sensitivity for endogenous and exogenous glucocorticoids can be influenced by many factors and conditions, including not only altered uptake, altered steroid-binding proteins in the plasma, or alterations in the 11βHSD activity, but also the GR-expression level, the ligand binding affinity of GR, the ability of GR to bind DNA, or by competition for DNA-binding with other inflammatory transcription factors [88]. Several methods have been used to study glucocorticoid sensitivity: in vivo by, e.g., very low dose (0.25 mg) dexamethasone suppression tests, and in vitro, e.g., by glucocorticoid receptor transactivation or transrepression activity using bioassays or gene expression profiles [89, 90].

### Individual Variations in Glucocorticoid Sensitivity and Their Consequences for Body Composition and Mental Status

A number of acquired diseases are currently linked to differences in tissue sensitivity to glucocorticoids, such as HIV (which is associated with glucocorticoid hypersensitivity) [91, 92] and inflammatory states such as rheumatoid arthritis, osteoarthritis, inflammatory bowel disease, and asthma (associated with resistance to glucocorticoids) [91].

However, also certain polymorphisms in the GR gene (NR3C1) have been identified that influence glucocorticoid sensitivity, of which a small number have been studied most intensively: *Tth111*I, ER22/23EK, N363S, Bcl*I*, and GR-9β [93].

The Bcl*I* polymorphism, associated with an increase in glucocorticoid sensitivity, was linked to increased central obesity and BMI, as well as other parameters that are related to the metabolic syndrome, including insulin resistance and increased blood pressure [94, 95]. Roerink et al. recently found that the Bcl*I* polymorphism is associated with an adverse cardiometabolic risk profile in patients in long-term remission of Cushing syndrome [57]. The exact mechanism that may cause the Bcl*I* polymorphism to increase glucocorticoid sensitivity has not been unraveled.

The N363S polymorphism is also related to an increase in glucocorticoid sensitivity and has been associated with a higher BMI and increased LDL cholesterol in elderly [96]. However, the positive relationship of N363S and BMI has not consistently be observed [97, 98••]. This polymorphism has been shown to lead an increased transactivational capacity of the GR in vitro [99] and to altered gene expression [90].

As an opposite to N363S and Bcl*I*, the combined ER22/23EK polymorphism has been shown to lead to a mild glucocorticoid resistance, both in vivo as in vitro studies [99, 100]. In line with a long-term decreased GC action, we previously observed sex-specific changes in body composition. Male carriers of this polymorphism have more muscle mass, increased muscle strength, and are taller; female carriers have a smaller waist circumference [101]. Furthermore, it has been associated with higher insulin sensitivity and lower fasting insulin, CRP, and LDL-cholesterol levels [98••].

The GR-9β polymorphism has no effect on GR transactivation but seems to decrease transrepression activity of the GR. This type of mild GC resistance may reduce the immunosuppressive actions of glucocorticoids and has been associated with an increased incidence of coronary heart disease [93].

The abovementioned polymorphisms can be integrated in five different haplotypes of the GR-gene [93]. Recently, these haplotypes were studied in 12,552 individuals of the Lifelines cohort study [102]. Haplotype 4, containing the N363S polymorphism, showed an increase in MetS in young adult males. Interestingly, this haplotype increased the odds for MetS specifically in patients with a low education status. It is conceivable that in the context of stressful circumstances, such as poverty or unemployment, the effects of the stress hormone cortisol may cause extra harm in individuals carrying the N363S variant. Thus, genetic variations in the GR-gene may influence the metabolic phenotype, which seems also influenced by environmental factors, and may contribute to individual vulnerability for the biological effects of stress.

Genetic variations in the HPA axis could play a role in stress-related mental disorders, such as post-traumatic stress disorder [103] and mood disorders [104]. Previous studies have also specifically focused on the link between GR polymorphisms and depression. As to the variants associated with increased glucocorticoid sensitivity, most studies have focused on the Bcl*I* polymorphism and found that depressive patients were more likely to be Bcl*I* variant carriers [105–107]. On the other hand, the relative glucocorticoid-resistant ER22/23EK SNP was also more frequently observed in patients with a major depressive disorder [105, 108], which concurs with the common observation of relative glucocorticoid resistance (with biochemical assessments) in depressive patients. One other specific example is FKBP5, a cochaperone in the heat shock protein 90-steroid receptor complex, which not only is induced by GR activation, but also inhibits the GR, thus creating an ultra-short intracellular negative feedback loop [87]. High FKBP5 levels are known to decrease GR signaling by alteration of the GR complex. Several common genetic variants within this GR signaling pathway have been linked to depression [109]. These studies show that either increased or decreased exposure to GCs at the molecular level, as determined by an individual genetic makeup, seems to be involved in major depressive disorder.

## The Interplay Between the Stress System and Obesity

**Fig. 1:**
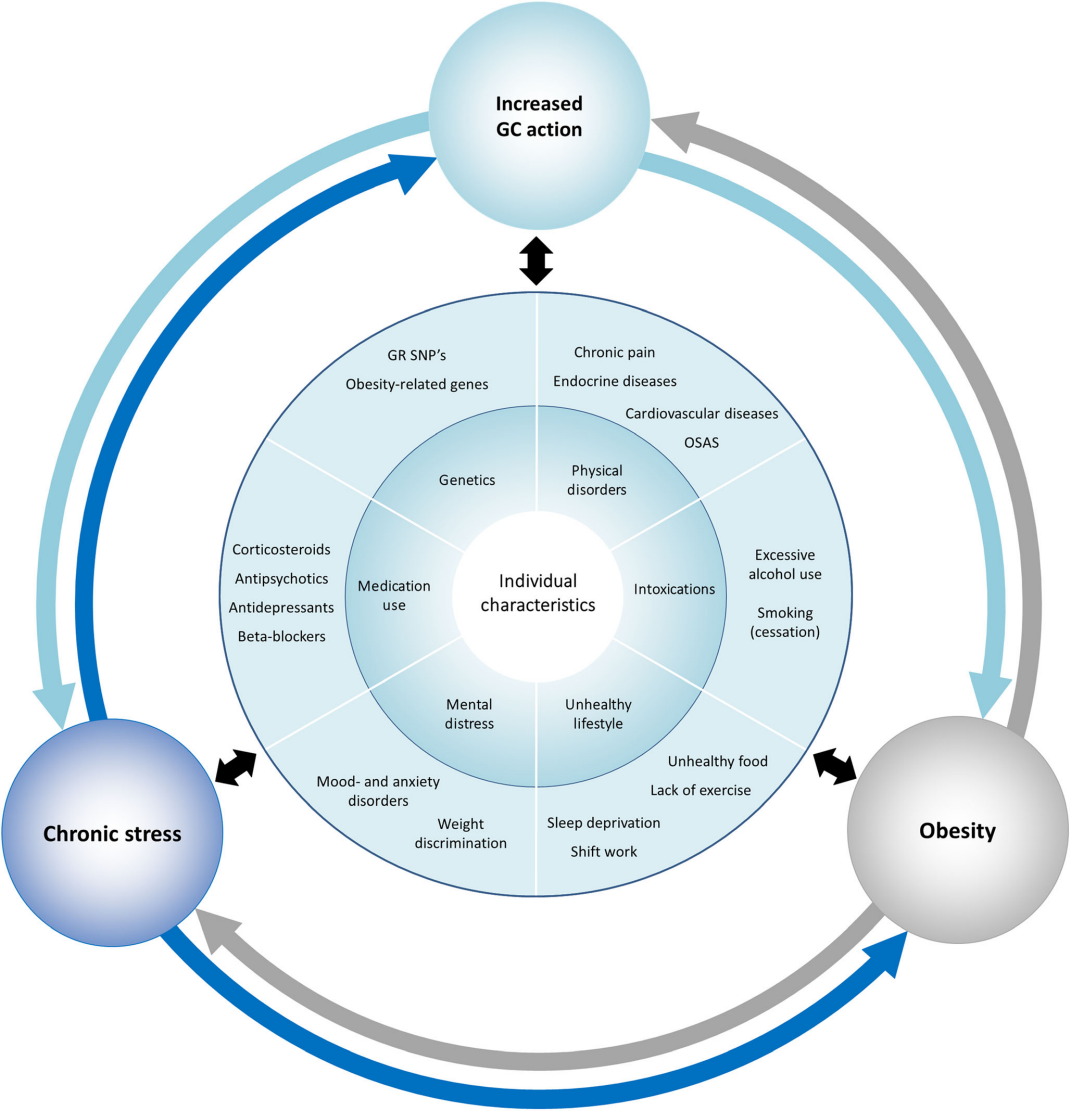
Conceptual model of the interplay between the stress system and obesity. Various individual characteristics are proposed to play a role in initiating a vicious circle of increased activation of the stress system (both by perceived chronic stress as well as increased net glucocorticoid effect by either endogenous or exogenous GCs) and obesity in a bidirectional manner. Abbreviations: GC, glucocorticoid; SNP, single nucleotide polymorphism; OSA, obstructive sleep apnea

Integrating the abovementioned aspects, a conceptual model for the interplay between the stress system and obesity in which individual patient characteristics play a central role has been proposed (Fig. 1). The end effects of stress with respect to weight gain can be accomplished in different ways due to the various properties of glucocorticoids. High levels of cortisol can, for example, increase appetite with a preference for “comfort food” and cause white adipose tissue to redistribute to the abdominal region [1•], which may ultimately lead to abdominal obesity [4]. Interestingly, it had been observed that glucocorticoids may decrease the sensitivity to adrenergic stimulation of brown fat [110, 111]. Furthermore, exogenous glucocorticoid administration increases the intrahepatic conversion of cortisone to cortisol thereby potentially contributing to the vicious circle [112]. This relationship between (chronic) stress and obesity mediated by increased glucocorticoid action may in some persons be greater by exposure to factors enhancing the stress response. Biological factors, such as carrying glucocorticoid sensitive GR gene variants, or a disrupted diurnal cortisol rhythm by decreased sleep and/or shift work, can potentially lead to higher glucocorticoid effects and thus make certain persons more prone to weight gain, and obesity. Moreover, the same holds for other environmental and behavioral factors, such as intake of food with high glycemic index, excessive alcohol use, and chronic pain, all possibly leading to increased cortisol levels and higher body weight. [71, 113].

On the other hand, obesity per se can also lead to increased chronic stress to varying degrees depending on certain individual characteristics. Persons experiencing, for example, weight stigma are known not only to experience more stress [114] but also to have higher long-term cortisol levels [30]. Additionally, persons with obesity are more likely to suffer from mental (e.g., depression) and physical disorders (e.g., OSAS, chronic pain due to weight load) which can in turn lead to chronic stress and/or higher cortisol levels. This can even be exaggerated by the use of certain medications indicated for obesity-related comorbidities, such as corticosteroids for arthrosis or asthma.

Thus, in this and many other ways, a vicious circle may be formed that maintains chronic stress, obesity, and increased GC action, leading to even more weight gain and/or impeding weight loss.

## Conclusions

Summarizing recent literature, it can be concluded that there are more susceptible individuals in the bidirectional relation between stress and obesity. This can be partly traced back to a third key player affecting stress and obesity: increased glucocorticoid action. The latter is influenced by individual GC sensitivity and altered levels of GCs, which have both been associated with body composition and mental disorders in recent studies.

Future studies may yield more insight into the vicious circle of obesity, stress, and increased GC action, which may lead to more, individualized treatment strategies that integrate obesity and stress.
